# Beyond the Brain-Vogt-Koyanagi-Harada Syndrome (VKH): A Rare Brain Eye and Ear (BEE) Syndrome Presenting initially as Aseptic Meningitis- a Neurologist Perspective

**DOI:** 10.4314/ejhs.v34i4.10

**Published:** 2024-07

**Authors:** Erum Shariff, Asma Khalaf Alzuabi

**Affiliations:** 1 Department of Neurology, College of Medicine, Imam Abdulrahman Bin Faisal University, Dammam, Saudi Arabia; 2 Department of Ophthalmology, College of Medicine, Imam Abdulrahman Bin Faisal University, Dammam, Saudi Arabia

## Abstract

**Background:**

The triad of central nervous system, hearing, and visual disturbances is an often encountered scenario. Vogt Koyanagi-Harada (VKH) is a rare syndrome affecting tissues with melanocytes and characterized by bilateral diffuse granulomatous uveitis, meningeal involvement, and hearing impairment. VKH is considered a rare cause of Brain Eye and Ear (BEE) syndrome.

**Case:**

We describe a case of a 32-year-old healthy lady who was admitted to neurology with the initial impression of aseptic meningitis. She had subacute onset of headache and fever, associated with blurring of vision and painful eye movements. Visual acuity 20/250 of the right eye and 20/80 of the left eye. Intra-ocular pressure measured 12 for the right eye and 14 for the left eye, and extraocular muscle movements were full Slit lamp examination showed a quite conjunctiva and clear cornea; however, there was an anterior chamber reaction of 2+ cells. The fundus exam showed mild vitritis with hyperemic disc swelling of both eyes and exudative retinal detachment bilaterally. Macular optical coherence tomography (OCT) demonstrated the presence of vitritis, pockets of subretinal fluids with bacillary layer detachment, and choroidal thickening. She was treated with steroids and mycophenolate mofetil with an excellent outcome.

**Conclusion:**

Early diagnosis has good outcomes and of crucial to prevent damage to the photoreceptors and subsequent poor visual outcomes. The presence of a distinctive expression in one BEE organ should prompt the appropriate investigations and multidisciplinary team involvement to avoid permanent vision loss.

## Introduction

Vogt Koyanagi-Harada (VKH) disease is a multisystem autoimmune disease that predominantly targets melanin-related antigens. The main characteristics of the disease are; bilateral diffuse granulomatous uveitis, meningeal irritation, hearing impairment, and vitiligo. It is an uncommon however treatable cause of visual impairment. Meningitis is one of the most common neurological presentations, nonetheless cranial neuropathies, encephalopathies, and myelitis have been reported in the literature (1). Neurological and auditory manifestations often precede the visual impairment posing a diagnostic dilemma. Meticulous examination for fundus abnormalities and subtle skin findings like poliosis may help recognize this disease at the bedside.

## Case Report

A 32-year-old lady was admitted with two weeks of acute onset of headache and fever. Headache was associated with blurring of vision and painful eye movements. However, there was no redness or discharge from the eyes reported. She was initially started on antibiotics and corticosteroids to cover meningitis. She had a low-grade fever and the rest of the examination was unremarkable except for reduced visual acuity bilaterally. She refused lumber puncture (LP) initially and after 4 days LP was performed under fluoroscopic guidance. CSF shows; WBCs 2, Protein 36.9, Glucose 78, Albumin 20.5, Gram Stain negative.

**Figure 1:**
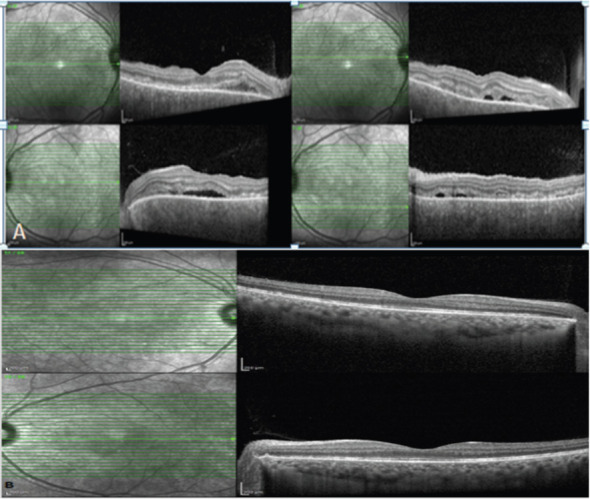
Spectral-domain optical coherence tomography of the right and left eye at the time of ophthalmology presentation and 3 days following meningitis treatment; showing vitreous, pockets of subretinal fluids with bacillary layer detachment, and choroidal thickening:
Spectral-domain optical coherence tomography of the right and left eye one month following the treatment showed resolution of the vitreous, subretinal fluid with the restoration of the photoreceptors and retinal pigment epithelium and residual thickening of the choroid.Spectral-domain optical coherence tomography of the right and left eye one month following the treatment showing resolution of the vitritis, subretinal fluid with restoration of the photoreceptors and retinal pigment epithelium and residual thickening of the choroid.

Ophthalmology consultation on day 3 of meningitis therapy revealed visual acuity of 20/250 in the right eye and 20/80 in the left. Intra-ocular pressure measured 12 for the right eye and 14 for the left eye, and extraocular muscle movements were full. Slit lamp examination showed a quite conjunctiva and clear cornea; however, there was an anterior chamber reaction of 2+ cells. The fundus exam showed mild vitritis with hyperemic disc swelling of both eyes and exudative retinal detachment bilaterally. Macular optical coherence tomography (OCT) demonstrated the presence of vitritis, pockets of subretinal fluids with bacillary layer detachment, and choroidal thickening (Fig. 1A).

**Figure 2:**
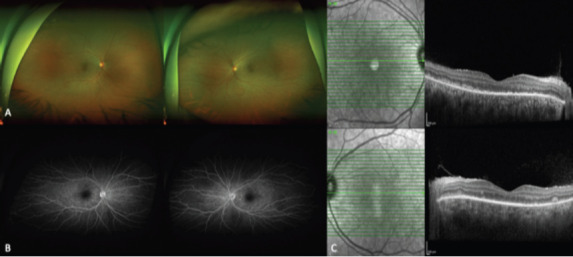
Five days following meningitis treatment:
Wide-field fundus photos of the right and the left eye showing hyperemic disc with right eye superotemporal and inferotemporal pockets of subretinal fluid, and left eye nasal and inferior pockets of subretinal fluidWide-field fundus fluorescein angiography of the right and the left eye shows hyperfluorescent spots representing choroidal granulomas with mild pinpoint leakage and hot discSpectral-domain optical coherence tomography of the right and left shows improvement of vitreous and subretinal fluid and nodularity and thickening of photoreceptors and retinal pigment epithelium

Based on these findings, the impression was initial onset acute VKH disease. Further infectious workups for tuberculosis, syphilis, and HIV were all negative. Fundus fluorescein angiography, showed hyperfluorescent spots representing choroidal granulomas with mild pinpoint leakage and hot disc (Figure 2). Repeated OCT showed surprising improvement of vitritis and subretinal fluid, and noted nodularity and thickening of photoreceptors and retinal pigment epithelium before the initiation of pulse steroid therapy. This unexpected improvement was explained by the meningitis therapy regimen the patient was receiving, as it included 10 mg intravenous dexamethasone every 6 hours (Figure 2).

Intravenous methylprednisolone pulse therapy of 1g for three days was initiated along with oral mycophenolate mofetil 1g every 12 hours. The clinical exam showed further improvement in the vitritis and resolution of the subretinal fluid. The patient was discharged on oral mycophenolate mofetil 1g every 12 hours along with a tapering regimen of oral prednisolone therapy with a starting dose of 70mg/day and tapering by 10mg every ten days till the 20mg/day dose milestone, after that the tapering schedule to be continued by reducing 5mg every ten days. The patient was kept as well on alfacalcidol 0.25mcg/day, calcium carbonate 600mg/day, and pantoprazole 40mg/day as supplements for steroid-related side effects.

Regular follow-ups and regular lab monitoring were continued. At the most recent follow-up, two months following the discharge, the examination showed visual acuity of 20/30 for both eyes, a quiet anterior segment exam with no cellular reaction. The Posterior segment exam of both eyes revealed a clear vitreous, healthy disc, and flat retina with some residual pigmentary changes. OCT macula of both eyes showed resolution of the vitritis and subretinal fluid, with the restoration of the photoreceptors and retinal pigment epithelium and residual thickening of the choroid (Figure 1 B).

## Discussion

The work-up and diagnosis of patients presenting with auditory, visual, and neurological symptoms can be challenging although certain investigation findings are highly diagnostic. Differential diagnosis of VKH syndrome includes Behcet's disease, and sarcoidosis especially when the neurological presentation precedes the systemic manifestations. Typical ophthalmological findings in these different diseases are important to reach the pertinent diagnosis. VKH shows the classic manifestations of bilateral optic nerve head inflammation and exudative retinal detachment, Behcet's disease shows anterior segment inflammation with classically mobile hypopyon, posteriorly it manifests as retinitis, occlusive retinal vasculitis, and inflammatory vein occlusion, while sarcoidosis has granulomatous anterior uveitis, intermediate uveitis, retinal vasculitis, and rarely choroidal granuloma.

An autoimmune process triggered by some viruses is assumed to be responsible for the pathogenesis of this syndrome. As it is observed in certain ethnicities viz Asian, Hispanics, and Native Americans and specifically with Human Leucocyte Antigen (HLA) haplotypes (HLA DRB1*0405 and HLA DQ4), a genetic influence is possibly suspected to be involved in the pathogenesis of disease and usually affects patients between the ages of 20 and 50 years. 1 The antigenic targets for VKH are the tyrosinase family proteins and gp100, against which specific T cells are directed (2)._This explains the predilection for melanocyte-rich organs. These CD41 T cells may be triggered by an infectious agent; this process is facilitated by the presence of certain HLA types, including HLADRB1* 0405 and HLA DR 4/DR53, which may unmask cryptic self-epitopes on the surface of melanocyte-specific proteins and activate T cells that would otherwise be silent.

The clinical course of VKH syndrome can be divided into prodromal, acute uveitis, convalescent, and chronic recurrent stages. Neurological and auditory involvement occurs in the prodromal stage. Ocular involvement occurs in acute uveitis, convalescent, or chronic recurrent stage, whereas skin involvement occurs in the convalescent stage (3).

The neurological manifestations of VKH syndrome consist of headache, meningismus, and occasionally, focal neurological signs. It's of note that these neurological and auditory symptoms are not always present and many VKH patients present with ocular signs without preceding any neurological or auditory symptoms.

History of ocular trauma or surgery must be absent. Our patient met the criteria for “incomplete VKH” given the absence of integumentary findings. She presented with a recent onset new headache and fever initially and then progressed to have a visual impairment. Recently two cases were reported with incomplete and complete VKH associated with headache and highlighted that the headache associated with blurred vision is a red flag (4). Multisystem involvement poses the diagnostic conundrum. A case of VKH was reported with seronegative rheumatoid arthritis preceding the diagnosis (5). Retrospective data of three patients with VKH was analyzed and found that three patients were first diagnosed with different degrees of meningitis (6). For these patients, the prognosis was good, and brain parenchymal damage was rare. However, the mechanism of brain parenchyma damage remains unclear and may be associated with enhanced intracranial osmotic pressure.

When neurological features precede ocular involvement, we should consider the incomplete VKH possibility. VKH syndrome commonly presents to ophthalmologists but neurologists may occasionally encounter this disease. Diagnostic difficulty occurs as neurological features often precede ocular involvement and many patients have an incomplete syndrome. VKH syndrome is a rare and serious vision-threatening disease that could result in permanent visual loss if treated late. It is important to consider VKH syndrome in any patient with aseptic meningitis if tests for common infectious and inflammatory diseases are negative.

The case report shows the importance of early involvement of a multidisciplinary team in the absence of apparent ocular abnormalities/trauma explaining symptoms and negative meningitis workup, prompt diagnosis by eye exam should be done to ensure early diagnosis and treatment to prevent permanent visual loss. Steroids are the mainstay of treatment for the disease with the addition of other immunosuppressants to avoid steroid-related long-term complications.
